# Paternal under-nutrition programs metabolic syndrome in offspring which can be reversed by antioxidant/vitamin food fortification in fathers

**DOI:** 10.1038/srep27010

**Published:** 2016-06-03

**Authors:** Nicole O. McPherson, Tod Fullston, Wan Xian Kang, Lauren Y. Sandeman, Mark A. Corbett, Julie A. Owens, Michelle Lane

**Affiliations:** 1 Discipline of Obstetrics and Gynaecology, School of Medicine, Robinson Research Institute, University of Adelaide, South Australia, 5005, Australia; 2 Freemasons Centre for Men’s Health, University of Adelaide, South Australia, 5005, Australia; 3 Discipline of Paediatrics, School of Medicine, Robinson Research Institute, The University of Adelaide, South Australia 5005, Australia; 4 Monash IVF Group, Melbourne, Victoria, 3168, Australia

## Abstract

There is an ever increasing body of evidence that demonstrates that paternal over-nutrition prior to conception programs impaired metabolic health in offspring. Here we examined whether paternal under-nutrition can also program impaired health in offspring and if any detrimental health outcomes in offspring could be prevented by micronutrient supplementation (vitamins and antioxidants). We discovered that restricting the food intake of male rodents reduced their body weight, fertility, increased sperm oxidative DNA lesions and reduced global sperm methylation. Under-nourished males then sired offspring with reduced postnatal weight and growth but somewhat paradoxically increased adiposity and dyslipidaemia, despite being fed standard chow. Paternal vitamin/antioxidant food fortification during under-nutrition not only normalised founder oxidative sperm DNA lesions but also prevented early growth restriction, fat accumulation and dyslipidaemia in offspring. This demonstrates that paternal under-nutrition reduces postnatal growth but increases the risk of obesity and metabolic disease in the next generation and that micronutrient supplementation during this period of under-nutrition is capable of restoring offspring metabolic health.

Worldwide, under-nutrition is a common affliction that affects 870 million adults and children, accounting for 55% of global morbidity and mortality, with pregnancy, perinatal and childhood periods being particularly sensitive^1^. Maternal under-nutrition both before and during pregnancy can restrict fetal growth and can program offspring for increased risks of noncommunicable diseases (NCDs)^2^, such as increased risks of cardiovascular disease and type II diabetes in children; as documented for the children born during the Dutch famine for example^3^. Data from Sweden also implicates a paternal origin for under-nutrition based programming, with grandpaternal famine associated with obesity and cardiovascular disease in grandchildren^4^. However the impact on the first generation and whether this relationship between a grandfather’s food restriction and their grandchild’s metabolic disorders is causal remains unclear. Other models of paternal programming (i.e. over-nutrition) report impairment to the health of both first and second generation offspring, manifesting as an increased risk of metabolic syndrome and subfertility^5^,^6^,^7^. This paternal programming from over-nutrition may act, in part, by oxidative stress in both testes and sperm^8^,^9^,^10^ that ultimately leads to adverse embryo, fetal and placental growth^11^,^12^,^13^. Here we examined the effects of paternal under-nutrition on growth, metabolic homeostasis and gene expression in offspring, and whether oxidative stress (as measured by reactive oxygen species; ROS) in sperm is a mediating factor. We induced under-nutrition in male mice by restricting dietary food intake (DR) to 70% of *ad libitum* controls (CD) for 17 weeks (Table S1) and examined the effects in juvenile and adult offspring.

Under-nutrition and its associated comorbidities have been combatted in developing countries through micronutrient supplementation and food fortification^14^, typically by supplementing folate, iron and zinc in undernourished children and women of reproductive age^14^. Many of these micronutrients act as antioxidants, known to reduce oxidative lesions in sperm^15^. We therefore examined whether micronutrient supplementation of undernourished males could reverse or prevent any adverse outcomes to sperm and subsequent offspring. To achieve this we fortified their food during DR with vitamins/antioxidants (DRVO, Table S1). All males were then mated with normal weight *ad libitum* fed female mice, who were allowed to litter, and then their offspring were assessed.

## Results

### Founder diet restriction reduces body weight, impairs sperm function and global methylation, and impacts preimplantation embryo development

DR and DRVO reduced body weight, adipose depots (gonadal, omental, renal and dorsal) plasma leptin and corticosterone, but improved glucose and insulin tolerance in founder males, compared with CD (P < 0.05, Table 1). There was no effect of DR on sperm count, motility or morphology, however DR increased sperm ROS and oxidative DNA lesions (P < 0.05, Table 2), which were restored by DRVO (Table 2). Further assessment of sperm function included it’s capacity to bind to the zona pellicuda, which demonstrated a reduction in the numbers of sperm bound to oocytes following IVF using sperm from DR males compared with CD males (P < 0.05, Table 2). Again sperm-oocyte binding was restored by DRVO. However supplementation of a CD cohort with antioxidant and vitamins (CDVO) did not affect any parameter measured that has previously been associated with inducing an offspring phenotype including body composition measures (weight or adiposity; Table 1), glucose or insulin tolerance or sperm parameters (count, motility, ROS levels, or sperm binding ; Table 2) and therefore this cohort was not investigated further.

For embryo development DR increased 2-cell cleavage rates compared with CD (P < 0.05, Table 2), but by 78 h of culture also displayed delayed blastocyst development (P < 0.05, Table 2), both of which were restored by DRVO (Table 2). By 96 h of culture no change in blastocyst development or blastocyst cell number was seen between treatments (P > 0.05, Table 2). DR did not alter mating frequency, time to mate, gestational length or litter size, but did reduce fertility (decreased pregnancy rates post-successful mating) and the proportion of female offspring (P < 0.05, Table 2). Pregnancy rates and female/male offspring ratios were normalised in DRVO males (P < 0.05, Table 2).

As altered levels of dietary folate have previously been shown to alter the methylation status of sperm^16^ and our VO group included the addition of folic acid (the synthetic form of folate) we further assessed the global methylation (5 mC) by immunocytochemistry in sperm. 5mC levels in sperm were low in all treatment groups (0–3% of sperm which stained positive, Table 2). Interestingly 5mC were reduced in DR compared with CD (P < 0.05, Table 2, Fig. S1) and was partially restored by DRVO (p = 0.06, Table 2, Fig. S1).

### Diet restricted fathers reduce offspring postnatal weight, growth, alter adiposity accumulation and size

**Figure 1:**
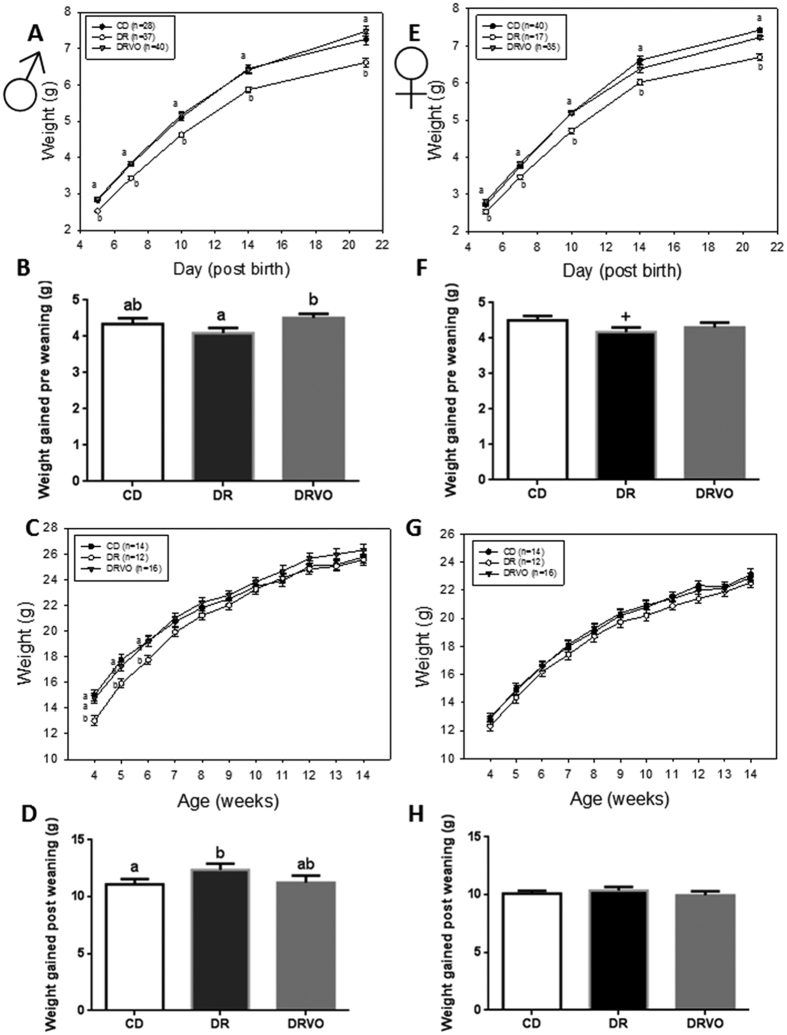
Vitamin and antioxidant supplementation of under nourished fathers restores neonatal size and growth. (**A**) Early postnatal growth of male offspring until weaning (21 days old). (**B**) Total weight gained in male offspring from day 5 until day 21. (**C**) Weights of male offspring from 4 weeks until 14 weeks. (**D**) Male offspring total amount of weight gained post weaning from 4 weeks of age until 14 weeks. (**E**) Early postnatal growth of female offspring until weaning (21 days old). (**F**) Total weight gained in female offspring from day 5 until day 21. (**G**) Weights of female offspring from 4 weeks until 14 weeks. (**H**) Female offspring total weight gained post weaning from 4 weeks until 14 weeks. Data represents 28 male and 40 female offspring from 7 litters representing 6 founder males for CD, 37 male and 17 females from 6 litters representing 6 founder males for DR and 40 male and 35 female offspring from 8 litters representing 8 founder males for DRVO. Statistical significance was determined by repeated measures ANOVA, with founder ID added as a random factor to both pre and post weaning weights and mother ID added as a random factor to pre weaning weights, to adjust outcomes for offspring generated from the same founder and same mother. Data is expressed as mean ± SEM. Different letters denote significance at P < 0.05; ^+^denotes significance to CD and DRVO at p = 0.06.

**Figure 2:**
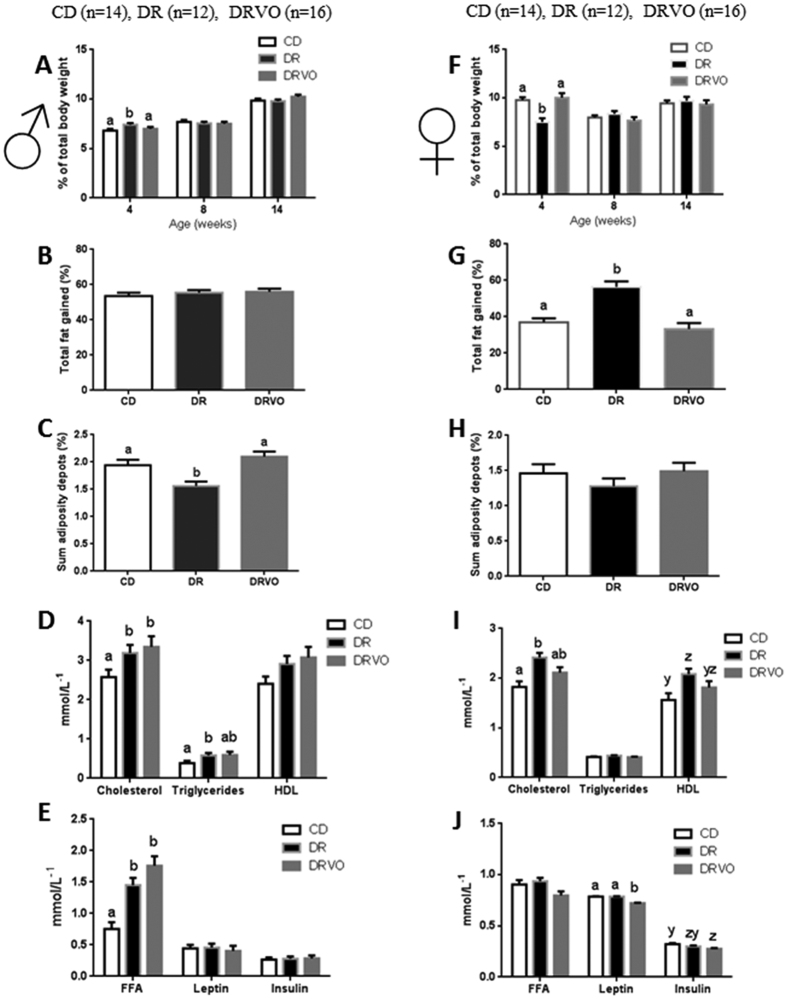
Vitamin and antioxidant supplementation of under nourished fathers restores male and female offspring adiposity and circulating lipids, insulin and leptin in female offspring. (**A**) Total adiposity as a percentage of body weight in male offspring as measured by DEXA at 4, 8 and 14 weeks of age. (**B**) Total adiposity gained (4 to 14 weeks) in male offspring as a percentage of fat mass at 14 weeks as determined by DEXA. (**C**) Sum of adipose depots (omental, gonadal, renal and dorsal) as a percentage of body weight at 15 weeks for male offspring. (**D**) Fasted serum lipids in male offspring at 15 weeks. (**E**) Fasted free fatty acids, leptin and insulin levels in male offspring at 15 weeks. (**F**) Total adiposity as a percentage of body weight in female offspring as measured by DEXA at 4, 8 and 14 weeks. (**G**) Total adiposity gained (4 to 14 weeks) in female offspring as a percentage of fat mass at 14 weeks as determined by DEXA. (**H**) Sum of adipose depots (omental, gonadal, renal and dorsal) as a percentage of body weight at 15 weeks for female offspring. (**I**) Fasted serum lipids in female offspring at 15 weeks. (**J**) Fasted free fatty acids, leptin and insulin levels in female offspring at 15 weeks. Data represents 14 females and 14 males from 7 litters generated from 6 founders for CD group, 12 females and 12 males from 6 litters generated from 6 founders for DR group, and 16 females and 16 males from 8 litters generated from 8 founders for the DRVO group. Statistical significance was determined by repeated measures ANOVA, with founder ID added as a random factor for adiposity data and a general linear mixed model with founder ID added as a random factor for all other measures to adjust outcomes for offspring generated from the same founder. Data is expressed as mean ± SEM. Different letters denote significance at P < 0.05.

**Figure 3:**
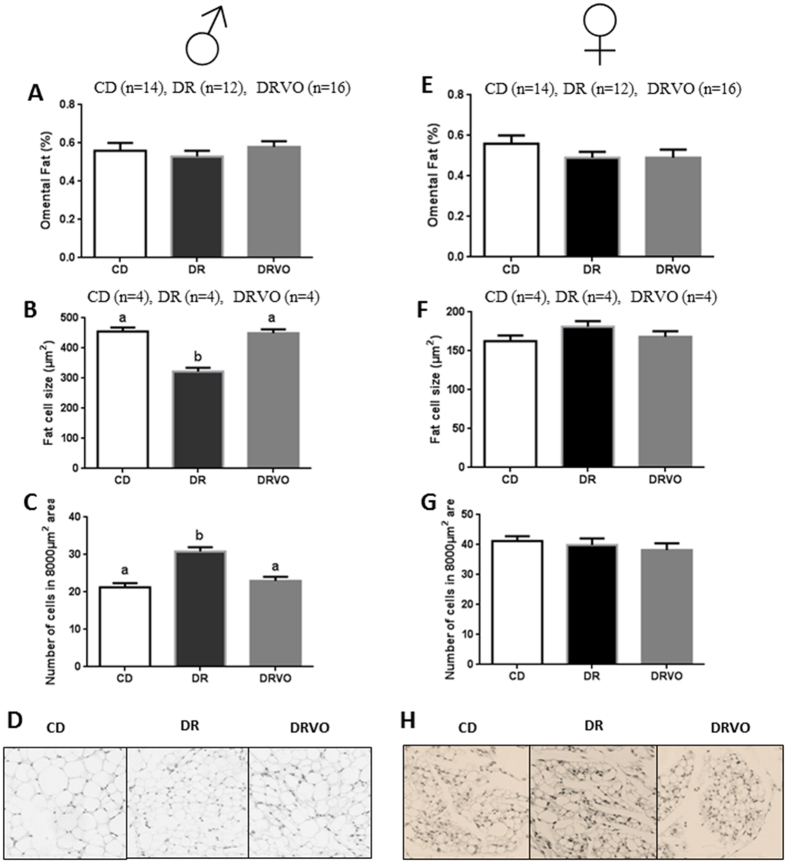
Vitamin and antioxidant supplementation of under nourished fathers restores male offspring adipocyte number and size. (**A**) Male offspring omental fat as a percentage of total body weight at 15 weeks. (**B**) Male offspring adipocyte cell size of omental fat at 15 weeks. (**C**) Male offspring adipocyte cell number at 15 weeks. (**D**) Male offspring omental fat cell size representative pictures for each founder treatment (40x). (**E**) Female offspring omental fat as a percentage of total body weight at 15 week PM. (**F**) Female offspring fat cell size of omental fat at 15 weeks. (**G**) Female offspring fat cell number at 15 weeks (**H**) Female offspring omental fat cell size representative pictures for each founder treatment (40x). Omental fat percent of total body weight represents 14 females and 14 males from 7 litters generated from 6 founders for CD group, 12 females and 12 males from 6 litters generated from 6 founders for DR group, and 16 females and 16 males from 8 litters generated from 8 founders for the DRVO group. Adipocyte number and size represents 4 males and 4 females from 4 litters generated from 4 founders for each treatment group. Statistical significance was determined with a general linear mixed model, with founder ID added as a random factor to adjust outcomes for offspring generated from the same founder. Data is expressed as mean ± SEM. Different letters denote significance at P < 0.05.

Founder DR reduced male and female offspring weights postnatally (d5–d21; P < 0.05, Fig. 1A,D) and reduced pre-weaning weight gain (Fig. 1B,F). Sons showed slower growth until 6 weeks of age (P < 0.05, Fig. 1C, Table S2) followed by a ‘catch up growth’ phase, resulting in similar weight to CD by 7 weeks (P < 0.05, Fig. 1C,D). Prenatal and early postnatal growth restriction is known to be associated with changes in body composition, including increased adiposity later in life^17^. Interestingly, founder DR increased relative (to total body weight) fat mass in sons at 4 weeks (P < 0.05, Fig. 2A), but this dissipated by 8 weeks of age (Fig. 2A,B, Table S2). At 15 weeks of age sons had reduced fat mass in selected central and peripheral fat depots in both absolute and relative terms (P < 0.05, Fig. 2C, Table S4) and reduced adipocyte cell size in omental fat depot (Fig. 3A) (P < 0.05, Fig. 3B,D), while the number of adipocytes was increased, indicating greater cell density (P < 0.05, Fig. 3C,D). At this same age DR sons had increased circulating lipids (total cholesterol and triglycerides (P < 0.05, Fig. 2D) and free fatty acids (FFA, P < 0.05, Fig. 2E). Interestingly, DR sons had improved insulin sensitivity at 14 weeks (p < 0.05, Fig. S3B,C).

In contrast, DR daughters had reduced relative fat mass at 4 weeks of age (P < 0.05, Fig. 2F, Table S3), however accumulated more body fat as they matured (P < 0.05, Fig. 2G) resulting in normal adult adiposity (Fig. 2F,H, Tables S3 and S5), with omental adipocyte cell size and density not altered at 15 weeks (Fig. 3E–H). DR daughters had increased circulating total and HDL cholesterol (P < 0.05, Fig. 2I) at 15 weeks, despite normal adiposity, again suggesting impaired lipid metabolism and homeostasis. DR daughters also had reductions in fasting and fed plasma glucose at 8 weeks (p < 0.05, Fig. S2D) and at 14 weeks (p < 0.05, Fig. S3E) respectively, but no alterations in glucose tolerance or insulin tolerance at these ages (Fig. S3).

### Vitamin and antioxidant supplementation to diet restricted fathers reverse postnatal weights and adult adiposity

Other paternal nutritional manipulations that initiate paternal programming implicate oxidative stress in sperm as a potential mediator^5^,^6^,^18^. Thus we examined if paternal supplementation with vitamins/antioxidants known to reduce sperm ROS^15^ during DR in fathers (DRVO) could rescue offspring phenotypes. Founder DRVO normalised both male and female offspring weight, growth and adiposity both pre-weaning (P < 0.05, Fig. 1A,B,E,F) and post-weaning (P < 0.05, Fig. 1C,D,G,H), to that of offspring born to CD. Both DRVO sons and daughters had restored adiposity accumulation and adipocyte phenotypes, compared with DR offspring (P < 0.05, Figs 2 and 3, Tables S2, S3, S4 and S5). Founder DRVO did not normalise the elevated plasma total cholesterol, triglycerides or FFA in adult sons (Fig. 2E,D). However, circulating lipids were improved in DRVO daughters, with reductions of plasma total and HDL cholesterol comparable to CD offspring (Fig. 2I). Furthermore, DRVO daughters had reduced plasma leptin and insulin compared to CD offspring (P < 0.05, Fig. 2J). Similar to DR founder DRVO improved insulin sensitivity at 8 weeks of age in sons (P < 0.05, Figs S2A, S3C), although this was not seen by 14 weeks (Fig. S2B,C); the age when DR son’s improved insulin sensitivity was significant. Although there were no changes to insulin and glucose tolerance in DRVO daughters at 8 and 14 weeks of age, they maintained lower fasting and fed glucose levels prior to the 8 week GTT and 14 week ITT (Fig. S3D,E), similar to that of DR daughters.

### Founder oxidative status in sperm correlates with male offspring postnatal growth

In normal weight men supplementation of vitamins/antioxidants has been shown to be beneficial to sperm health^19^,^20^,^21^ with specific reductions in ROS mediated sperm DNA lesions in a sub-fertile population^15^. Consistent with this, we saw a greater proportion of DR males with increased sperm oxidative DNA lesions, which was reduced in DRVO (P < 0.05, Table 2) and a positive correlation between sperm 8-OH-dG levels and sperm ROS (P < 0.05, Table 2). Sperm DNA 8-OH-dG, a marker of oxidative lesions, correlated negatively with son’s postnatal weight, independent of founder diet (p < 0.01, d5 −0.260; d7 −0.304; d10 −0.350; d14 −0.424; d21 −0.374 and weight gained −0.374), however this was not evident in daughters.

### Diet restriction in fathers alters gene expression in offspring in a sex specific manner with partial normalisation by vitamin and antioxidant supplementation

Changes to the gene expression of pancreas have been reported for other models of paternal nutritional programing of offspring phenotype^7^,^22^, so the effect of DR and DRVO were investigated in the pancreas of offspring.

When offspring where analysed regardless of sex, paternal DR altered the expression of *Vaultrc5*, *Arl1*, and *Casp9* in pancreas compared with CD (FDR < 0.05), but DRVO did not affect their expression compared with DR (Supplementary Table S6).

Male offspring fathered by DR males had reduced expression of *Gm20594* and *Lars2* in pancreas compared with CD (FDR < 0.05), but again DRVO did not affect their expression compared with DR (Supplementary Table S6).

**Figure 4:**
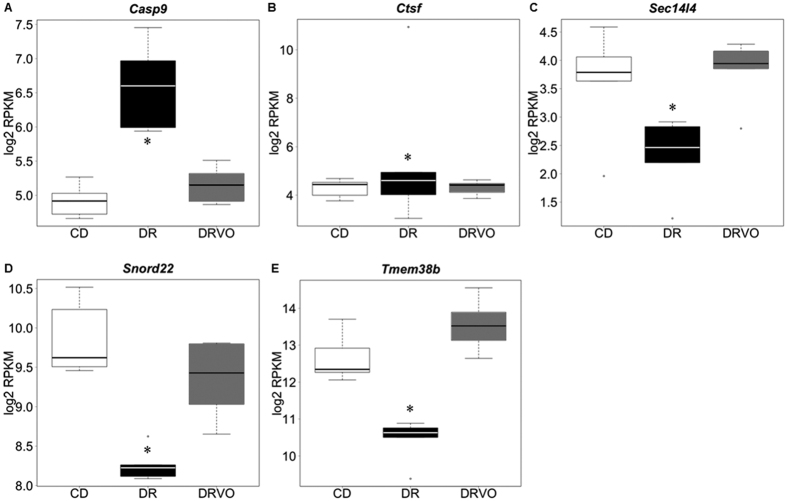
Vitamin and antioxidant supplementation of under nourished fathers partially restores gene expression the pancreas from female offspring. Box plots represent the gene expression by RNA sequencing of the pancreas from 6 female offspring each selected from a different litter per paternal diet group. Differential expression was determined between groups by first fitting a generalised linear model to these data incorporating diet of the father, sex of the mouse and RIN scores as covariates. Differences between groups with fathers fed under different diet conditions were determined by log rank test, correcting for multiple testing with the false discovery rate method. Data is expressed as log2 of the mean from the 6 samples of normalised reads per kilobase per million reads (RPKM) per transcript ± SEM. *Significantly different to the pancreatic expression of that gene in female offspring born to CD founder (FDR < 0.05; p < 0.001).

Female offspring fathered by DR males had alteration to 14 genes in the pancreas compared with CD (FDR < 0.05; Supplementary Table S6). Interestingly 5/14 were normalised by DRVO (*Casp9*, *Ctsf*, *Sec14l4*, *Snord22*, *Tmem38b*) to an abundance equivalent to CD (Fig. 4), whereas the remaining 8/14 were not. An analysis of enrichment of gene ontology terms in differentially expressed genes^23^ in female offspring due to paternal DR revealed a pattern of genes involving developmental processes. Specific gene ontologies that were enriched in differentially expressed genes include apoptosis, organogenesis, neuronal cell fate/differentiation, pattern specification, cell projection organisation (lamellipodium) and G-coupled receptor signaling.

When differential gene expression was analysed regardless of sex, in male offspring only, and in female offspring only 1 (*Vaultrc5*), 2 (*Gm20594*, *Lars2*), and 4 (*Snord15b*, *Snord15a*, *Snord17*, *Eif5a*) genes, respectively, were down-regulated by DR (DR and DRVO fathers) regardless of micronutrient supplementation (Supplementary Table 6; blue highlight). Conversely micronutrient supplementation (DRVO) dysregulated 5 genes (3 up-regulated – *miR-2137*, *Gm20594*, *miR-5109*; 2 down-regulated – *3110062M04Rik*, *Dmp1*) in pancreas of female offspring regardless of diet restriction (Supplementary Table 6; green highlight); but this phenomena was not seen when analysis was performed regardless of offspring sex nor in male offspring only.

It must be noted that there was a greater number of adult pancreas genes with altered expression in DRVO compared with CD, than DR vs CD for all groups. For example when data was analysed regardless of offspring sex 4 genes were altered by DRVO, 3 of which were not altered by DR alone, compared with CD. In male offspring 7 genes were altered by DRVO, 5 of which were not altered by DR alone, compared with CD. Finally in female offspring DRVO altered the expression of 60 genes; 56 were not also altered by DR, compared with CD.

## Discussion

Paternal under-nutrition of specific dietary components including low protein diet and folate insufficiency, or in utero under nutrition in males has previously been shown to alter fertility and increase offspring susceptibility to cardiovascular disease and metabolic syndrome^16^,^24^,^25^. Our study confirms these findings in a mouse model of 70% caloric restriction, designed to mimic the environment of a developing country. The subsequent assessment of the effects to fertility and offspring health was also extended to determine if these phenotypes could be reversed through vitamin and antioxidant food fortification to our under nourished founder males. We show for the first time that reduced offspring weight, dyslipidemia and adipose accumulation, and altered pancreas gene expression that resulted from paternal under-nutrition can be restored by vitamin and antioxidant food fortification in fathers. Additionally we show that diet restriction increases oxidative sperm DNA lesions and reduces global sperm methylation which were also somewhat normalised by food fortification.

70% diet restriction with or without vitamin and antioxidant supplementation reduced founder male body weight, adiposity and plasma leptin and corticosterone levels while improving glucose and insulin regulation similar to other rodent models of caloric restriction^26^,^27^. Interestingly there was no effect of DR on commonly assessed fertility parameters (sperm count, motility or morphology) used in fertility clinics to assess male fertility^28^. However, DR did decrease the number of sperm that could bind to an MII oocyte, indicating a potential decrease in post ejaculation sperm maturation (capacitation and hyperactivation) both necessary requirements for sperm binding^29^. Sperm binding also utilizes glucose^30^, and the reduced circulating glucose concentrations in DR males may have also contributed to their reduced sperm binding. Interestingly this reduced sperm binding from DR fathers did not alter bound sperm’s capacity to fertilize oocytes, with DR males displaying increased 2-cell cleavage rates, indicating that their reduced sperm binding was likely of no immediate biological significance. But it must be noted that by 78 h of embryo culture DR males did have a reduction in the number of blastocysts that developed. Increased levels of sperm ROS and oxidative DNA lesions *in vitro*, as seen here in DR males, has previously been demonstrated to reduce blastocyst formation without altering 2-cell cleavage rates or the total cell number if blastocysts^18^.

While there were no changes to mating rates as evident by a vaginal copulation plug, the number of plugged females that went on to make a successful litter were reduced for DR males. In humans it has been shown that delayed on time embryo development is associated with reduced embryo implantation^31^, and therefore the delayed on time blastocyst development seen at 78 hours of culture may have contributed to reduced embryo viability. Interestingly the sex ratio of pups was altered by DR with DR fathers producing more male offspring, which is consistent with the model of paternal protein restriction^24^. This suggests DR may have altered the molecular composition of sperm, consistent with other experimental paradigms of paternal programming^6^,^24^,^32^. The unchanged litter size and sperm function suggests that paternal DR alters offspring sex ratio, potentially by altered proportions of X/Y chromosome bearing sperm impairing individual function^33^. Given that the gene expression due to DR was most changed in the pancreases of adult female offspring at 15 weeks of age, compared with male offspring, perhaps X chromosome bearing sperm are more sensitive to the changes that enact the transmission. Interestingly, 5 of the 14 genes dysregualted in the pancreas of female offspring sired by paternal diet restriction were restored to the same abundance as female’s born to control fathers by micronutrient supplementation during diet restriction. Although it must be noted that more genes were altered by the combination of diet restriction with micronutrient supplementation than were rescued, two distinct subsets of dysregulated genes were unique to either diet restriction or micronutrient supplementation, and that the altered expression of these genes in offspring tissue as a result of paternal nutritional programming has not been previously reported. It is enticing to speculate that the alteration of the expression of these 5 genes due to paternal diet restriction and their normalisation by micronutrient fortification during diet restriction suggests that they may be altered/restored by the same mechanism that also causes/restores offspring phenotype – albeit manifesting only in female offspring. For example the up-regulation of *Casp9* may imply an increased apoptosis within the pancreas and might partly explain some of the metabolic defects observed in female offspring. The dysregulation of the other 4 genes have also been associated with human and animal pathologies, warranting further validation/investigation; including mutations in *Tmem38b* that are associated with reduced bone mass, and reduced *Ctsf* abundance associated with aberrant neural cell growth. Furthermore, the gene expression results highlight the importance of analysing male and female offspring as separate entities.

Similar to previous mouse models of paternal nutrient restriction (protein and folate)^16^,^24^ as well as paternal over nutrition^6^,^7^ we observed sex specific effects to offspring metabolic health. DR reduced both male and female neonatal and post natal weights up until weaning, followed by a period of ‘catch up growth’ in early adulthood. This phenotype follows a similar pattern to that of small for gestational age children who are born small and then display this catch up growth. Prenatal and early postnatal growth restriction is known to be associated with increased incidence of metabolic health problems later in life^17^. Male offspring of DR fathers had increased fat mass at 4 weeks of age, which manifested as increased adipocyte cell numbers without an increased in fat mass by 15 weeks of age, similar to that of male offspring born to fathers exposed to low protein diets^24^. Thus DR sons may have an increased propensity to deposit fat if challenged by an obesogenic environment later in life^34^. This double hit phenomenon of (1) parental peri-conception challenge followed by (2) adult offspring environmental exposure is seen in many developing countries where under-nutrition and obesity co-exist^35^,^36^. By 15 weeks of age male offspring had increase plasma lipid concentrations (cholesterol, triglycerides and FFA) suggesting impaired lipid metabolism and homeostasis. A similar phenotype has been reported by paternal protein restriction, which increased hepatic cholesterols in offspring, presumably due, in part, to altered expression of transcripts (*Hmgcr, Pmvk, Sqle* and *Sc4mol*) which target SREBP; a major transcriptional regulator of cholesterol metabolism^37^. Female offspring of DR fathers had reduced fat mass at 4 weeks of age, however, gain more adipose tissue throughout life, so that by 15 weeks of age they demonstrated similar adiposity to that of control offspring. This period of increased fat deposition coincided with abnormal plasma lipid profile. Whether the underlying mechanism driving their elevated total and HDL cholesterol and increased rate of fat accumulation will result in obesity later in life is unclear. However, the DR daughter phenotype is reminiscent of the phenotype observed in Senegalese girls, who as a result of under-nutrition and stunted growth as children, have increased accumulation of relative body fat during puberty, with greater deposition of subcutaneous fat in central and upper regions of their bodies at fifteen years of age^38^. The reduced female offspring plasma glucose concentration observed here due to paternal DR has been previously shown, whereby founder male rodents were fasted for 24h prior to mating that led to reduced serum glucose in offspring by 6 weeks of age^39^. Interestingly we saw no effects to glucose or insulin tolerance testing from our offspring sired by DR fathers. Whether additional and more marked changes would emerge as offspring age as seen in a setting of paternal protein restriction (at 22 weeks of age)^24^, or even as a secondary consequence to their abnormal lipid profiles at 15 weeks, remains to be determined. Of note, in some experimental paradigms of paternal nutritional manipulation, systemic phenotypic changes in metabolism emerge only later in life in aged offspring^6^,^7^,^24^ with only changes at the molecular level observed in early life^7^,^22^.

One hypothesis for the generation and transmission of paternally-mediated pathologies to offspring, which involves the incomplete repair of sperm DNA lesions at fertilisation, has been suggested by Aitken and colleagues^40^. Sperm are highly susceptible to ROS as they have lost most of their cytoplasmic scavenging enzymes during maturation (spermiogenesis)^41^,^42^, their plasma membrane contains large quantities of polyunsaturated fatty acids which are particularly susceptible to ROS^43^, and when released into the epididymis they have left the protection of the blood testes barrier. At fertilisation, the oocyte has a limited capacity to repair these oxidative DNA lesions, and therefore incomplete or aberrant repair of these oxidative DNA lesions hold the potential to create mutations which could inhabit every cell of the subsequent embryo/offspring. This hypothesis is highly relevant to our findings, as increased ROS and oxidative DNA lesions were elevated in sperm of DR fathers, and could be a contributing factor to the impairments observed in their offspring phenotypes. The artificial induction of oxidative stress in mouse sperm by H_2_O_2_ also resulted in reduced postnatal growth and increased adiposity in both male and female offspring^18^, similar to the phenotypes reported for this DR model.

Interestingly all effects from DR on male sperm function, ROS levels, oxidative DNA lesions, embryo development, pregnancy establishment, sex specific effects on offspring metabolic health and some sex specific pancreas gene expression differences were normalised by antioxidant and vitamin food fortification of DR fathers. Micronutrient supplementation (vitamin E, vitamin C, zinc, folate, selenium) to infertile men has previously been shown to reduce seminal ROS levels after 3 months of antioxidant treatment^15^. It is therefore likely that the reductions to sperm ROS and oxidative DNA lesions result from the antioxidant supplementation. The epididymal endothelial ion transporters operate between the circulating blood and epididymial lumen^44^,^45^, allowing for ingested antioxidants to come into contact with the sperm during this important transitional step and to degrade free radicals, reducing the number of oxidative lesions. This suggests that improvements to offspring growth, adipose accumulation and lipid accumulation due to an undernourished father maybe reversed through reductions to sperm ROS via antioxidant and vitamin food fortification.

Other potential mechanisms for paternal programming also include epigenetic changes to sperm (small non coding RNAs, methylation, and histone changes) and seminal fluid composition. This is evidenced by chemically induced site specific changes to sperm DNA methylation within the male pronucleus being associated with increased post-implantation pregnancy loss^46^, aberrant abundance of sperm microRNAs programming offspring phenotypes^47^, and altered seminal fluid composition inducing pregnancy loss and programming metabolic disease in offspring^48^. Relevant to this study, models using low folate diets (folate is a known methyl donor) demonstrate changes to sperm methylation at the promoters of genes important for developmental and metabolic processes^16^, and that in zebra fish the sperm methylome is inherited by the embryo at fertilisation^49^. Interestingly, DR males had a reduced number of sperm nuclei positive for 5mC methylation staining, which tended towards normalisation by antioxidant and vitamin food fortification (i.e. p = 0.06). However, it should be noted that the abundance of 5mC detected in mouse sperm by immunocytochemistry was low and detection of 5mC was likely limited due to the tight packaging of mouse DNA around protamines (99%), even the after DNA denaturing and condensation steps used in the protocol. Further studies assessing site specific methylation patterns of developmental, metabolic, and paternally imprinted genes and whether changes are caused by DR and normalized by antioxidant and vitamin food fortification is warranted.

We have demonstrated that paternal under-nutrition impairs pancreatic gene expression and lipid homeostasis in offspring, with sex specific effects on adiposity, fat accumulation that suggest an increased risk of obesity in response to an obesogenic environment or with aging. Furthermore vitamin and antioxidant food fortification in fathers, normalises the expression of some genes in the pancreas of female offspring, rescues ROS and somewhat normalised global methylation patterns in sperm, normalised postnatal growth and associated adiposity in male and female offspring, with sex specific improvements to circulating lipids in females and insulin sensitivity in males, albeit transiently. This proof of concept study identifies the undernourished father as a novel source of the global disease burden and as a modifiable target in its prevention through food fortification, that likely acts in concert with the detrimental maternal under-nutrition pathways. Whether similar benefits will be observed in humans and the direct mode of transmission warrants further investigation.

## Methods

### Animal Ethics

This study was carried out in strict accordance with the Australian code of practice for the care and use of animals for scientific purposes. The use and care of all animals used in the study was approved by the Animal Ethics Committee of The University of Adelaide.

### Founder Males and Diets

C57BL6 male mice (5 weeks old) were fed either a control diet (CD, n = 10), containing 6% fat, 19% protein and 64.7% carbohydrate, a restricted diet (DR, n = 10) or a restricted diet with vitamin and antioxidant supplementation (DRVO) for 17 weeks (Table S1). The diet for DRVO was supplemented with vitamin C, vitamin E, folate, lycopene, zinc, selenium and green tea extract for the vitamin intervention. For diet restriction C57Bl6 male mice were restricted to 70% of *ad libitum* intake of the control diet, using a step-down protocol, with restriction to 90% for the first week, 80% in the second week, 70% in the third week then maintained at 70% *ad libitum* intake for a further 14 weeks (22 weeks of age). A control diet with antioxidant supplementation (CDVO) cohort was also included, but not mated. Animals were individually housed for the entire study and had water supplied at all times.

### Founder Body Composition

Founder body weights were recorded weekly. At 22 weeks of age adiposity (gonadal adiposity, omental adiposity, retroperitoneal adiposity, peritoneal adiposity and dorsal adiposity), liver and pancreas were collected, weighed and performed blinded by the same individual.

### Founder Glucose Tolerance Test (GTT) and Insulin Tolerance Test (ITT)

At 20 weeks of age a GTT was performed after 6 h of fasting by intra-peritoneal (IP) injection of 2 g/kg of 25% D-glucose solution. ITT was performed at 22 weeks of age during a fed state by IP injection of 1.0 IU of human insulin (Actapid®, Novo Nordisk, Bagsvaerd, Denmark). Tail blood glucose concentrations were measured using a glucometer (Hemocue, Angelholm, Sweden) at time points 0 (pre-bolus basal), 15, 30, 60 and 120 min. Data were expressed as mean blood glucose concentration per group as area under curve (AUC) for glucose and area above the curve (AAC) for insulin.

### Founder Sperm Count, Motility and Morphology

Sperm count, motility and morphology were assessed in accordance with WHO guidelines^28^ (at least 200 sperm counted for each sample). Sperm count was determined by counting on a haemocytometer. Sperm motility was assessed blinded under a light microscope classifying 200 sperm per animal as either progressive motile, non-progressive motile or immotile. Motility was then expressed as percentage of total motile sperm (progressive motile + non progressive motile sperm). Sperm morphology was assessed blinded on samples fixed with methanol:acetone (3:1) and stained with haematoxylin and eosin. For identifying sperm morphology individual sperm were classified as normal, tail defect (bent tails and twisted tails) or head defect (large heads, small heads and deformed heads) as per^9^. Morphology is expressed as percentage normal forms.

### Founder Sperm Binding

Sperm binding to the zona pellucida were performed as described in Bakos *et al*.^50^. Briefly mature cumulus-enclosed oocytes (COCs) were collected from 4-week old Swiss female mice 12 h following super ovulation with intraperitoneal injections of PMSG and hCG administered 48 h apart. Sperm (1 × 10^4^/ml) were co-incubated with COCs in G-IVF for 1 hr at 37 °C, 6% CO_2_ and 5% O_2._ At 1 hr post insemination, sperm binding was determined by counting the number of bound sperm to the oocyte under phase contrast microscopy. At least 10 oocytes were analysed per sperm sample.

### Intracellular Sperm Reactive Oxygen Species (DCFDA)

Intracellular ROS levels were assessed in progressively motile sperm by incubation with 1μM DCFDA (2′,7′-dichlorodihydrofluorescein diacetate, Invitrogen) for 20 min at 37 °C as per^51^. Sperm were then washed twice and imaged using a fluorescent microscope with photometer attachment. Intracellular ROS levels were expressed as average fluorescent units (FU).

### Sperm Immunocytochemistry

Oxidative stress induced DNA lesions in sperm was assessed by oxidative guanine DNA (8-OH-dG) stained sperm modified from^52^ while global sperm methylation was assessed by 5-methylcytosine (5mC). Sperm were fixed to polylysine slides with 3:1 ratio of methanol to acetone for 5 mins and then permeabilised for 45 min in 0.5% Triton-X-100. Slides were then washed in PBS and sperm nuclei decondensed for 60 min at 37 °C in 1M HCL, 10 mM Tris buffer containing 5 mM dithioretiol. Sperm nuclei were then denatured in 6M HCL/0.1% Triton-X-100 for 45 min at room temperature followed by neutralisation in 100 μM Tris HCL. Sperm slides were then incubated in either 1:100 mouse Anti-8 Hydroxyguanosine antibody [N45.1] (ab48508, Abcam, Cambridge, UK) or 1:00 mouse Anti-5mC (39649, Active Motif, Carlsbad, USA) in 10% donkey serum overnight at 4 °C followed by PBS washes and incubation in 1:100 Biotin-SP-conjugated AffiniPure F(ab’)_2_ fragmented donkey anti-mouse IgG (H + L) (715-065-150, Jackson ImmunoResearch, Baltimore, PA, USA) at room temperature for 2 h. Following PBS washes sperm slides were then incubated in 1:100 Cy3 conjugated streptavidin (016-160-084, Jackson ImmunoResearch) for 1.5 h at room temperature followed by nuclear counter staining with Hoechst. Slides were loading in glycerol/prolong gold solution and imaged under epifluorescent microscopy. For 8-OH-dG staining a positive control was included where by sperm were treated prior to fixation with 1500 μM H_2_O_2_ to induce sperm oxidative DNA lesions. For both 8-OH-dG and 5mC staining a negative control was added where the primary antibodies were omitted from the reaction. For 8-OH-dG staining 200 sperm per animal were assessed as either positive for 8-OH-dG staining or negative (Fig. S4) and this expressed as a percentage. For the 8-OH-dG positive control 99% of sperm were positively stained for 8-OH-dG while no sperm stained positive in the negative control. As there were very low levels of 5mC present in mouse sperm, 200 sperm per animal were viewed and classified as either positive for 5mC staining or negative.

### Embryo Collection

3–4 week old C57BL6 female mice who were fed standard chow were given an intraperitoneal (IP) injection of 5 IU Pregnant Mare’s Serum Gonadotropin (PMSG) (Folligon, Invervet, Bendigo, Australia), followed 48 h later with an IP injection of 5 IU human Chorionic Gonadotropin (hCG) (Pregnyl, Organon, Sydney, Australia) to induce ovulation as per^53^,^54^. Following the hCG injection female mice were individually placed with a founder male at 13 weeks of age (8 weeks on restricted diets). Successful mating was assessed the following morning by the presence of a vaginal plug. At 22–24 h post hCG female mice were humanly killed by cervical dislocation and, cumulus enclosed zygotes were collected and placed in MOPS (Vitrolife) at 37 °C and denuded of cumulus cells by 1 min incubation with 0.5 mg/ml hyaluronidase. Zygotes were washed twice in MOPS and once in G1 medium before culture in G1 media.

### Embryo Culture

Embryos were cultured in groups of 10 in 20 μl drops of either G1 (Vitrolife) under paraffin oil (Merck, New Jersey, USA) for the first 48 h at 37oC in 5% O2, 6% CO2 and 89% N2. Embryos were then washed and cultured in G2 (Vitrolife) for a further 48 h to the blastocyst stage. Blastocyst development was scored at 20 h culture (cleavage), 42 h culture (compaction), 78 h culture (early blastocyst) and 96 h culture (late blastocyst) was scored at 96 h of culture (late blastocyst). Development post 20 h was expressed as percentage of on time embryos as a proportion of 2-cell cleavage. All embryo culture dishes were prepared 4 h prior to embryo culture to allow for gassing and temperature equilibration.

### Blastocyst Cell Number and Differentiation

Allocation of cells to the trophectoderm (TE) and inner cell mass (ICM) in the blastocyst, was performed at 96 h of culture as previously described^55^. Briefly, blastocysts were placed into 0.5% pronase (Sigma) at 37 °C until the zona pellucida dissolved, before incubation in 10 μM of 2,4,6-trinitrobenzenesulfonic acid (TNBS, Sigma) at 4 °C for 10 minutes. Blastocysts were transferred into 0.1 mg/μl anti-dinitrophenyl- BSA (anti-DNP, Sigma) for 10 minutes at 37 °C and then placed in guinea pig serum (Sigma) with PI (Sigma) for 5 minutes at 37 °C. Finally, embryos were placed in bisbenzimide (Sigma) at 4 °C overnight. The following day the embryos were washed through 100% ethanol, mounted in glycerol and visualized using a fluorescence microscope.

### Mating to Produce F1 Offspring

Founder males were mated with normal weight naturally cycling C57BL6 females to generate F1 offspring. At 15 weeks of age (10 weeks on restricted diets), each male was allocated 2, 8–9 weeks old female to mate for a maximum period of 5 days. Both males and females were given access to ad libitum CD food during the natural mating (120 h) to limit under nutrition to the mother. Successful mating was assessed the following morning by the presence of a vaginal plug. Females who had successfully been mated were removed and fed ad libitum standard chow whilst the founder males returned to their treatment group. On day 14 of pregnancy females were individually housed prior to birth. Females were monitored for birth from 18 days gestation and birth date and litter numbers were recorded. Pups were tattooed on day 5 and weighed on days 5, 7, 10, 14 and 21. On day 21 post birth, 2 males and 2 female offspring were sampled randomly per litter, weaned, ear tagged and allocated to share housing (5/offspring/per sex/per cage) until 15 weeks of life. Offspring were fed ad libitum standard chow routinely provided by the animal house and weighed weekly. 14 females and 14 males were sampled from 7 litters generated from 6 founders for CD group, 12 females and 12 males sampled from 6 litters generated from 6 founders for DR group, and 16 females and 16 males samples from 8 litters generated from 8 founders for the DRVO group.

### F1 Offspring Body Composition

Pre weaning body weights were recorded on days 5, 7, 10, 12, 14 and 21 post birth and identification of individual pups was determined by a foot pad tattoo. Post weaning offspring received an identifiable ear tag and body weights were recorded up until 15 weeks of age. At 8 weeks and 14 weeks of age offspring underwent a dual-emission X-ray absorptiometry machine (DEXA) (Piximus, Ge Lunar, Wisconsin, USA) as previously described in^56^ to determine, bone, lean mass and adiposity body composition. At 15 weeks of age offspring underwent a full post mortem where adiposity (gonadal adiposity, omental adiposity, retro peritoneal adiposity, peritoneal adiposity and dorsal adiposity), liver, kidneys and pancreas were collected, weighed and performed blinded by the same individual.

### F1 Offspring GTT and ITT

At 8–9 weeks and 13–14 weeks offspring underwent repeated measures for glucose and insulin challenges respectively as described above (For ITT females were given a bolus of 0.75 IU of human insulin while male offspring were given a bolus of 1.0 IU). A 5 day break was given to each mouse between each test at each time point.

### Metabolites and Hormone Analysis

At 22 weeks of age for founder males and 15 weeks of age for F1 offspring overnight fasting blood serum was collected post mortem by a cardiac puncture under anaesthetic with 2% Isoflurane(1-chloro-2,2,2-trifluoroethyldifluoromethylether, Veterinary Companies of Australia, Kings Park, Australia). Serum cholesterol, free fatty acids (FFA), glucose, triglycerides and high-density lipoprotein were measured on a Cobas Integra 400 plus automated sampler system (Roche, Basel, Switzerland). Serum insulin and leptin levels were measured by either an Ultra-Sensitive Mouse Insulin ELISA Kit (#90080, Crystal Chem Inc, Downer Grove, Illinois, USA) or Mouse Leptin ELISA Kit (#90030, Crystal Chem Inc, Downer Grove, Illinois, USA as per the manufacturer instructions.

### Histology of F1 Offspring Omental Adiposity

F1 offspring omental adiposity was fixed overnight in 4% paraformaldehyde and stored in 70% ethanol until further use. Omental adiposity was embedded in wax using standard methods and 7 μm sections were cut and heat fixed onto super frost slides. Each slide contained four 7 μm sections 50 μm apart. Slides were dewaxed, rehydrated in ethanol dilutions and stained with haematoxylin and eosin as per standard methods. Slides were mounted in DPX mounting media and imaged using a NanoZoomer slide scanner (Hamamatsu Photonics, Sunayama-cho, Naka-ku, Japan). The areas of 100 adipocytes from at least 5 different sections per animal were measured using NanoZoomer NDP viewer software. Adipocyte cell number was determined by counting the number of visible adipocytes in an 8000 μm^2^ square in 9 different areas of tissue across 2–3 sections. Adipocyte size and number was determined in 4 male and female offspring representative of 4 litters and 4 founder males.

### RNA Sequencing of offspring pancreases

RNA was extracted from 6 adult (15 weeks of age) male/female mouse pancreases per paternal diet treatment using a Qiagen RNeasy mini kit (Qiagen. Hilden, Germany). rRNA depletion and random hexamers were used to make cDNA libraries (New England Biolabs, Ipswich, Massachusetts, USA) which were then sequenced (50 bp single-end, stranded) on an Illumina HiSeq 2500 (Illumina, San Diego, California, USA) by the SA cancer genomic facility (IMVS, Adelaide, Australia). Sequence reads were mapped to the mm10 build of the mouse genome with tophat v2.0.9^57^ and read counts per gene calculated with htseq-count^58^. RNA-Seq data was analysed with the edgeR package from bioconductor^59^. Genes with counts per million model mapped reads of less than 1 were filtered out as were reads with multiple mapping locations and those not mapping to known features. Differential expression was determined between groups by first fitting a generalised linear model to these data incorporating diet of the father, sex of the mouse and RIN scores as covariates. Differences between groups with fathers fed under different diet conditions were determined by log rank test, correcting for multiple testing with the false discovery rate method^59^.

### Statistics

All data were expressed as mean ± SEM and checked for normality using a Kolmogorov-Smirnov test and equal variance using a Levene’s test. Statistical analysis was performed in SPSS (SPSS Version 18, SPSS Inc., Chicago, USA) with AUC and AAC calculated in GraphPad Prism (GraphPad Software, San Diego, USA). A p value < 0.05 was considered to be significant. Founder measures were analysed by a one way ANOVA with Bonferroni post hoc test. Embryology was assessed by a Fishers Exact test. Offspring post mortems, fat cell size and metabolites were assessed by linear mixed modelling with a Bonferroni post hoc test. Offspring weights, GTT, ITT and DEXA were analysed using a Repeated Measures ANOVA with Bonferroni post hoc test. In the models founder ID and mother ID were added as random effects to adjust for dependence in results between offspring from the same father and same mother. Replicate was fitted as a covariate. Correlations were determined by a Pearson correlation for founder measures or a linear regression controlling for multiple observations for individual fathers for offspring measures.

## Additional Information

**How to cite this article**: McPherson, N. O. *et al*. Paternal under-nutrition programs metabolic syndrome in offspring which can be reversed by antioxidant/vitamin food fortification in fathers. *Sci. Rep.* **6**, 27010; doi: 10.1038/srep27010 (2016).

## Supplementary Material

Supplementary Information

## Figures and Tables

**Table 1:** Founder Male Body Composition and Metabolites.

	CD (n = 10)	CDVO (n = 10)	DR (n = 10)	DRVO (n = 10)
Total body weight (g)	30.8 ± 1.0^a^	30.2 ± 0.7^a^	23.3 ± 0.4^b^	22.3 ± 0.5^c^
Weight gained (g)*	13.7 ± 0.7^a^	13.2 ± 0.6^a^	7.1 ± 0.4^b^	5.8 ± 0.4^c^
Weight gained (%)*	76.2 ± 3.2^a^	73.2 ± 2.8^a^	40.9 ± 2.3^b^	33.5 ± 2.2^c^
*Adiposity*				
Gonadal fat (%)	3.47 ± 0.30^a^	3.23 ± 0.30^a^	2.04 ± 0.11^b^	1.58 ± 0.17^c^
Omental fat (%)	1.48 ± 0.03^a^	1.26 ± 0.09^a^	0.86 ± 0.09^b^	0.77 ± 0.06^b^
Renal fat (%)	1.41 ± 0.13^a^	1.39 ± 0.11^a^	0.69 ± 0.06^b^	0.53 ± 0.05^c^
Dorsal fat (%)	1.18 ± 0.09^a^	1.07 ± 0.12^a^	0.74 ± 0.06^b^	0.74 ± 0.05^b^
Sum of fat depots (%)	7.51 ± 0.52^a^	6.96 ± 0.55^a^	4.33 ± 0.28^b^	3.62 ± 0.25^c^
*Plasma Metabolites and hormones, glucose and insulin tolerance*				
Glucose (mmol/L^−1^)	10.95 ± 0.49^a^	10.11 ± 0.33^a^	7.16 ± 0.17^b^	6.76 ± 0.14^b^
Glucose tolerance (AUC, min.mmol)	847.1 ± 75.7^a^	676 ± 62.6^a^	259.4 ± 10.3^b^	307.8 ± 14.1^b^
Insulin (ng/mL^−1^)	0.31 ± 0.07	0.46 ± 0.13	0.26 ± 0.05	0.35 ± 0.07
Insulin tolerance (AAC, min.mmol)	228.2 ± 27.5^a^	264.4 ± 54.0^a^	390.3 ± 54.2^b^	389.0 ± 60.9^b^
Leptin (ng/mL^−1^)	6.67 ± 0.98^a^	6.46 ± 1.6^a^	1.58 ± 0.18^b^	1.17 ± 0.11^b^
Corticosterone (ng/mL^−1^)	202.6 ± 29.3^a^	155.4 ± 60.5^a^	160.6 ± 26.8^a,b^	128.6 ± 16.1^b^

Statistical significance was assessed by one way ANOVA, with a Bonferroni post hoc test.

^*^Weight gain was calculated by total body weight prior to diet restriction (5 weeks of age) and total body weight prior to post mortem (22 weeks of age).

Data are expressed as mean ± SEM.

Different letters denote significance at P < 0.05.

No superscripts are used where no statistical differences were found.

**Table 2:** Founder Male Fertility.

	CD (n = 10)	CDVO (n = 10)	DR (n = 10)	DRVO (n = 10)
*Sperm Measures*				
Sperm count (×10^6^)	20.7 ± 3.2	19.5 ± 2.4	23.3 ± 2.8	20.9 ± 1.6
Total sperm motility (%)	71.8 ± 2.8	67.4 ± 2.1	68.3 ± 4.4	64.7 ± 3.4
Normal sperm morphology (%)	33.4 ± 2.2	37.8 ± 2.5	36.9 ± 1.9	36.1 ± 2.5
Number of sperm bound to a MII oocyte	55.1 ± 1.2^a^	54.6 ± 2.0^a^	34.5 ± 1.2^b^	54.0 ± 1.1^a^
*Oxidative sperm DNA damage*				
8-OH-dG positive sperm (%)	4.0 ± 0.4 (2.1–5.1)^a^	0.9 ± 0.2 (0.0–2.0)^b^	6.3 ± 1.6 (2.5–11.1)^a^	4.4 ± 0.8 (2.0–6.4)^a^
Proportion of males with > 75% quartile sperm positive for 8-OH-dG	0^a^	0^a^	50^b^	0^a^
Sperm ROS levels (DCFDA) (FU)	472 ± 6 (413–536)	442.3 ± 34.5 (134–811)	490 ± 30 (224–1000)	478 ± 18 (254–670)
Proportion of sperm with > 75% quartile ROS	0^a^	16.7^a,b^	39^b^	17^a,b^
Correlation of sperm 8-OH-dG with sperm ROS	CCO = 0.578, p = *0.02*			
*Global sperm methylation*				
Sperm positive for 5mC (%)	0.79 ± 0.21^a^ (0–1.32)	1.66 ± 0.58^a^ (0.73–3.19)	0.38 ± 0.07^b^ (0–0.54)	1.13 ± 0.47^a,&^ (0.36–3.43)
*Embryo Development*				
2-cell cleavage (20 h culture) (%)	72.3^a^	72.2^a^	86.9^b^	76.9^a,b^
8-cell/compacting (42 h culture) (%)	77.2^a^	75.2^a^	70.0^a^	84.3^b^
Exp blast developed (78 h culture) (%)	84.5^a^	90.7^a^	65.7^b^	82.9^a^
Total blast developed (96 h culture) (%)	96.1	94.1	94.7	94.2
*Embryo cell numbers*(*96* *h culture*)				
Total cell number	55.0 ± 2.2	52.5 ± 2.1	56.2 ± 4.0	50.5 ± 1.7
Trophectoderm cell number	45.4 ± 2.0	42.2 ± 1.8	46.4 ± 3.7	41.4 ± 1.6
Inner cell mass cell number	9.6 ± 0.5	10.3 ± 0.5	9.8 ± 0.9	9.1 ± 0.4
*Mating and litter size*				
Mating rate (%)	96.3		100	93.1
Pregnancy rate (%)	50.0^a^		25.0^b^	44.4^a^
Time to mate (days)	1.95 ± 0.30		2.04 ± 0.26	1.64 ± 0.26
Gestational length (days)	19.4 ± 0.2		19.1 ± 0.1	19.5 ± 0.2
Litter size	7.08 ± 0.45		7.13 ± 0.24	7.09 ± 0.32
Female offspring (%)	59^a^		31^b^	46^a,b^

Embryo development data represents at least 100 embryos from 6 fathers per treatment. Proportional embryo development data was analyzed by a Fisher’s exact test. Mating and litter data represents 7 litters generated from 6 founders for CD group, 6 litters generated from 6 founders for DR group and 8 litters generated from 8 founders for the DRVO group. Data was averaged for each founder then analyzed by a one way ANOVA, with a Bonferroni post hoc test. Mating rates were determined by the number of females with copulation plugs and pregnancy rates were calculated based on number of plugged females that produced a litter. Oxidative sperm DNA damage was assessed in founders who produced offspring.

Data are expressed as mean ± SEM.

FU = Fluorescent units.

CCO = correlation coefficient.

Number in brackets following oxidative sperm DNA damage and global methylation represent lowest and highest values for the group.

Different letters denote significance at P < 0.05.

^&^Different to DR at p = 0.06.

No superscripts are used where no statistical differences were found.
