# Supplementary material for: Paternal under-nutrition programs metabolic syndrome in offspring which can be reversed by antioxidant/vitamin food fortification in fathers
Source: Sci Rep. 2016 Jun 3;6:27010. doi: 10.1038/srep27010 (PMC4891691; doi:10.1038/srep27010)
Supplement: Supplementary Information [file srep27010-s1.pdf]

SUPPLEMENTARY DATA

- Tables S1-S6
- Figures S1-S4

Title: Paternal under-nutrition programs metabolic syndrome in offspring which can be reversed by antioxidant/vitamin food fortification in fathers

Authors: Nicole McPherson[1,2], Tod Fullston[1], Wan Xian Kang[1], Lauren Sandeman[1], Mark A Corbett[3], Julie A Owens[1], Michelle Lane[1,4]

Table S1: Composition of control diet (CD) and DR diet supplemented with vitamins and antioxidants (+VO)

**Ingredient (%)**	**CD (SF12-012)**	**+VO (SF12-013)**
Sucrose	34.1	34.1
Casein acid	19.5	19.5
Canola oil	6.0	6.0
Cellulose	5.0	5.0
Wheat starch	30.5	29.5
Vitamins/Minerals	4.9	4.9
Antioxidant premix		1.0
Selenium premix (4.4% Se)[a]		0.14
LycoRed (20% Lycopene as supplied)[a]		0.19
Vitamin E (50% m/m)[a]		1.35
Folic acid (90%)[a]		0.06
Zinc sulphate monohydrate (35.8% Zn)[a]		2.79
Green tea extract[a]		0.95
Sucrose[a]		94.53
Digestible energy (kJ/g)	16.1	16.1
Digestible energy from lipids	14.0	14.0
Digestible energy from protein	21.0	21.0

Note: [a] Percentage of total antioxidant added

No vitamin C added to CD. Mean *ad libitum* consumption of feed per 24 h was 3.44 ± 0.05 g (*n*=6 male mice measured for daily feed intake over a 10 week period). This intake was used to determine total energy intake and level of food restriction for diet restricted animals.

Table S2: Size and body composition of male offspring as determined by Dual-energy X-ray absorptiometry (DEXA)

	CD (n=14)	DR (n=12)	DRVO (n=16)
4 weeks			
Weight (g)	**16.0 ± 0.45[a]**	**13.9 ± 0.38[b]**	**16.3 ± 0.41[a]**
Length (cm)	**8.17 ± 0.09[a]**	**7.79 ± 0.07[b]**	**8.18 ± 0.09[a]**
Bone mass (g)	**0.19 ± 0.01[a]**	**0.16 ± 0.01[b]**	**0.19 ± 0.01[a]**
Bone mass (%)	1.31 ± 0.02	1.26 ± 0.02	1.26 ± 0.02
Lean mass (g)	13.5 ± 0.39	11.8 ± 0.32	13.7 ± 0.38
Lean mass (%)	91.7 ± 0.21	91.3 ± 0.18	91.8 ± 0.21
Fat mass (g)	**0.99 ± 0.04[ab]**	**0.91 ± 0.04[a]**	**1.03 ± 0.04[b]**
Fat mass (%)	**6.8 ± 0.19[a]**	**7.4 ± 0.18[b]**	**7.0 ± 0.20[ab]**
8 weeks			
Weight (g)	21.3 ± 1.0	21.2 ± 0.81	21.5 ± 0.96
Length (cm)	**8.9 ± 0.36[ab]**	**8.2 ± 0.29[a]**	**9.1 ± 0.34[b]**
Bone mass (g)	**0.33 ± 0.01[ab]**	**0.31 ± 0.01[a]**	**0.33 ± 0.01[b]**
Bone mass (%)	1.70 ± 0.02	1.61 ± 0.02	1.64 ± 0.02
Lean mass (g)	**17.3 ± 0.42[ab]**	**17.2 ± 0.34[a]**	**18.4 ± 0.40[b]**
Lean mass (%)	90.8 ± 0.15	90.9 ± 0.12	90.9 ± 0.15
Fat mass (g)	1.43 ± 0.04	1.41 ± 0.03	1.51 ± 0.04
Fat mass (%)	7.70 ± 0.20	7.54 ± 0.17	7.51 ± 0.20
14 weeks			
Weight (g)	25.5 ± 0.62	24.8 ± 0.50	26.1 ± 0.60
Length (cm)	**9.32 ± 0.07[ab]**	**9.25 ± 0.06[a]**	**9.48 ± 0.07[b]**
Bone mass (g)	0.37 ± 0.01	0.37 ± 0.01	0.38 ± 0.01
Bone mass (%)	1.64 ± 0.02	1.63 ± 0.02	1.61 ± 0.02
Lean mass (g)	20.1 ± 0.45	20.0 ± 0.36	20.7 ± 0.4
Lean mass (%)	88.7 ± 0.22	88.7 ± 0.18	88.3 ± 0.21
Fat mass (g)	**2.19 ± 0.06[ab]**	**2.17 ± 0.05[a]**	**2.36 ± 0.06[b]**
Fat mass (%)	9.84 ± 0.21	9.79 ± 0.17	10.24 ± 0.21
Total fat gained (%)	53.5 ± 1.9	55.4 ± 1.5	55.9 ± 1.8

Date represents 14 males from 7 litters generated from 6 founders for CD group, 12 males from 6 litters generated from 6 founders for DR group, and 16 males from 8 litters generated from 8 founders for the DRVO group. Statistical significance was determined by repeated measures ANOVA with founder ID added as a random factor. Total fat gained (%) was determined by the difference in fat (g) between 4 and 14 weeks as a proportion of fat mass (g) at 14 weeks.

Data is expressed as mean ± SEM.

Different letters denote significance at P<0.05.

Table S3: Size and body composition of female offspring as determined by DEXA

	CD (n=14)	DR (n=12)	DRVO (n=16)
4 weeks			
Weight (g)	13.0 ± 0.5	11.9 ± 0.7	13.3 ± 0.8
Length (cm)	7.84 ± 0.08	7.55 ± 0.11	7.94 ± 0.12
Bone mass (g)	0.16 ± 0.01	0.14 ± 0.01	0.16 ± 0.01
Bone mass (%)	1.23 ± 0.03	1.25 ± 0.04	1.22 ± 0.05
Lean mass (g)	11.4 ± 0.5	10.0 ± 0.7	11.5 ± 0.08
Lean mass (%)	**89.2 ± 0.3[a]**	**91.5 ± 0.4[b]**	**88.8 ± 0.5[a]**
Fat mass (g)	**1.21 ± 0.04[a]**	**0.80 ± 0.06[b]**	**1.27 ± 0.06[a]**
Fat mass (%)	**9.77 ± 0.31[a]**	**7.50 ± 0.41[b]**	**10.02 ± 0.47[a]**
8 weeks			
Weight (g)	20.1 ± 0.6	18.9 ± 0.8	20.5 ± 0.08
Length (cm)	8.63 ± 0.09	8.76 ± 0.12	8.70 ± 0.13
Bone mass (g)	0.30 ± 0.01	0.28 ± 0.02	0.31 ± 0.02
Bone mass (%)	1.75 ± 0.03	1.69 ± 0.05	1.77 ± 0.05
Lean mass (g)	15.5 ± 0.4	14.9 ± 0.6	15.7 ± 0.7
Lean mass (%)	90.5 ± 0.4	90.5 ± 0.6	90.3 ± 0.7
Fat mass (g)	1.33 ± 0.07	1.36 ± 0.09	1.30 ± 0.10
Fat mass (%)	7.97 ± 0.23	8.34 ± 0.31	7.66 ± 0.34
14 weeks			
Weight (g)	23.1 ± 0.5	21.6 ± 0.6	23.1 ± 0.7
Length (cm)	9.15 ± 0.06	9.25 ± 0.08	9.16 ± 0.09
Bone mass (g)	0.38 ± 0.01	0.36 ± 0.01	0.38 ± 0.02
Bone mass (%)	1.82 ± 0.04	1.83 ± 0.05	1.84 ± 0.06
Lean mass (g)	18.4 ± 0.4	17.6 ± 0.6	18.4 ± 0.6
Lean mass (%)	88.8 ± 0.3	88.7 ± 0.4	88.9 ± 0.4
Fat mass (g)	1.95 ± 0.07	1.88 ± 0.09	1.92 ± 0.10
Fat mass (%)	9.44 ± 0.29	9.71 ± 0.40	9.32 ± 0.43
Total fat gained (%)	**36.9 ± 2.2[a]**	**56.4 ± 3.0[b]**	**33.2 ± 3.3[a]**

Date represents 14 females from 7 litters generated from 6 founders for CD group, 12 females from 6 litters generated from 6 founders for DR group, and 16 females from 8 litters generated from 8 founders for the DRVO group. Statistical significance was determined by repeated measures ANOVA with founder ID added as a random factor. Total fat gained (%) was determined by the difference in fat (gm) between 4 and 14 weeks as a proportion of fat mass (gm) at 14 weeks.

Data is expressed as mean ± SEM

Different letters denote significance at P<0.05

Table S4: Adult male offspring body composition

Post mortem body composition	**CD (n=14)**	**DR (n=12)**	**DRVO (n=16)**
Total body weight units	22.8 ± 0.5	22.9 ± 0.4	24.0 ± 0.5
Gonadal Adiposity (%)	**0.84 ± 0.06[a]**	**0.63 ± 0.05[b]**	**0.97 ± 0.06[a]**
Renal Adiposity (%)	**0.19 ± 0.01[a]**	**0.13 ± 0.01[b]**	**0.20 ± 0.01[a]**
Dorsal Adiposity (%)	**0.36 ± 0.02[a]**	**0.26 ± 0.01[b]**	**0.34 ± 0.01[a]**
Sum of Adiposity deposits (%)	**1.94 ± 0.10[a]**	**1.56 ± 0.08[b]**	**2.10 ± 0.09[a]**
Liver (g)	0.93 ± 0.04	0.95 ± 0.03	1.02 ± 0.04
Liver (%)	4.07 ± 0.11	4.10 ± 0.08	4.27 ± 0.11
Pancreas (g)	**0.14 ± 0.01[ab]**	**0.13 ± 0.00[a]**	**0.15 ± 0.01[b]**
Pancreas (%)	0.59 ± 0.03	0.58 ± 0.02	0.62 ± 0.03
Kidneys (g)	0.29 ± 0.01	0.29 ± 0.01	0.30 ± 0.01
Kidneys (%)	1.28 ± 0.03	1.28 ± 0.03	1.26 ± 0.03
Soleus (mg)	8.73 ± 0.53	7.96 ± 0.40	8.67 ± 0.48
Vastus lateralis (mg)	145.4 ± 5.3	140.1 ± 4.1	151.1 ± 4.8

Data represents 14 males from 7 litters generated from 6 founders for CD group, 12 males from 6 litters generated from 6 founders for DR group, and 16 males from 8 litters generated from 8 founders for the DRVO group. Statistical significance was determined by general mixed model with founder ID added as a random factor.

Data is expressed as mean ± SEM.

Different letters denote significance at P<0.05.

Table S5: Adult female offspring body composition

Post mortem body composition	**CD (n=14)**	**DR (n=12)**	**DRVO (n=16)**
Total body weight units	**20.5 ± 0.3[a]**	**19.4 ± 0.3[b]**	**19.8 ± 0.2[ab]**
Gonadal Adiposity (%)	0.50 ± 0.08	0.39 ± 0.07	0.53 ± 0.07*
Renal Adiposity (%)	0.11 ± 0.01	0.07 ± 0.01	0.10 ± 0.01
Dorsal Adiposity (%)	0.31 ± 0.03	0.31 ± 0.02	0.37 ± 0.03
Sum of Adiposity deposits (%)	1.46 ± 0.13	1.26 ± 0.11	1.49 ± 0.11
Liver (g)	0.80 ± 0.03	0.83 ± 0.03	0.85 ± 0.03
Liver (%)	3.91 ± 0.17	4.26 ± 0.11	4.30 ± 0.11
Pancreas (g)	0.13 ± 0.01	0.13 ± 0.01	0.12 ± 0.01
Pancreas (%)	0.65 ± 0.03	0.66 ± 0.03	0.61 ± 0.03
Kidneys (g)	**0.241 ± 0.005[a]**	**0.224 ± 0.004[b]**	**0.237 ± 0.004[ab]**
Kidneys (%)	1.18 ± 0.02	1.15 ± 0.02	1.20 ± 0.02
Soleus (mg)	6.91 ± 0.39	6.52 ± 0.33	7.30 ± 0.33
Vastus lateralis (mg)	119.5 ± 3.8	117.2 ± 3.2	119.6 ± 3.3

Date represents 14 females from 7 litters generated from 6 founders for CD group, 12 females from 6 litters generated from 6 founders for DR group, and 16 females from 8 litters generated from 8 founders for the DRVO group. Statistical significance was determined by general mixed model with founder ID added as a random factor.

Data is expressed as mean ± SEM.

Different letters denote significance at P<0.05.

*different to DR at p=0.06.

Supplementary Table S6. Differential pancreatic gene expression in adult offspring (n=6 at 15 weeks of age per paternal dietray group from individual litters) sired by CD, DR, and DRVO Founder males (FDR<0.05; p<0.001). Log fold change has been calculated from normalised sequence counts per transcript. (A footnote explains abbreviations/shading used in this table)

Differential pancreatic gene expression analysis **regardless of offspring's sex**

Gene Symbol	Entrez ID	Full Gene Name	logFC	logCPM	LR	p Value	FDR
**CD vs DR F0**							
Vaultrc5	378472	vault RNA component 5	-0.82	5.68	28.92	7.55E-08	0.00046
Arl1	104303	ADP-ribosylation factor-like 1	1.05	4.33	22.97	1.65E-06	0.00502
Casp9	12371	caspase 9	1.07	5.66	19.94	7.98E-06	0.01620
**CD vs DRVO F0**							
Snord15b	449631	small nucleolar RNA, C/D box 14B	-0.99	7.49	28.29	1.05E-07	0.00064
Vaultrc5	378472	vault RNA component 5	-0.70	5.68	22.32	2.30E-06	0.00599
Zc3h15	69082	zinc finger CCCH-type containing 15	0.66	5.05	21.85	2.95E-06	0.00599
Banp	53325	BTG3 associated nuclear protein	-1.06	3.74	17.31	3.17E-05	0.04826
**DR vs DRVO F0**							
N/A							

Differential pancreatic gene expression analysis limited to only **male offspring**

Gene Symbol	Entrez ID	Full Gene Name	logFC	logCPM	LR	p Value	FDR
**CD vs DR F0**							
Gm20594	100463512	predicted gene, 20594	-1.91	8.58	19.06	1.26E-05	0.03696
Lars2	102436	leucyl-tRNA synthetase, mitochondrial	-2.73	14.07	18.46	1.73E-05	0.03696
**CD vs DRVO F0**							
P2rx1	18436	purinergic receptor P2X, ligand-gated ion channel, 1	1.09	5.70	20.47	6.04E-06	0.01577
Vaultrc5	378472	vault RNA component 5	-1.02	5.40	19.92	8.08E-06	0.01577
Tmem38b	52076	transmembrane protein 38B	-3.16	12.72	19.31	1.11E-05	0.01577
Lars2	102436	leucyl-tRNA synthetase, mitochondrial	-2.80	14.07	18.48	1.72E-05	0.01828
Frmd4b	232288	FERM domain containing 4B	1.08	7.37	16.83	4.09E-05	0.03100
Mir5109	N/A	N/A	-1.85	6.31	16.71	4.36E-05	0.03100
Gm20594	100463512	predicted gene, 20594	-1.75	8.58	15.64	7.66E-05	0.04667
**DR vs DRVO F0**							
N/A							

Differential gene expression analysis limited to only **female offspring**

Gene Symbol	Entrez ID	Full Gene Name	logFC	logCPM	LR	p Value	FDR
**CD vs DR F0**							
Casp9	12371	caspase 9	1.77	5.97	48.34	3.58E-12	1.91E-08
Snord15b	449631	small nucleolar RNA, C/D box 14B	-1.36	7.57	43.81	3.61E-11	9.64E-08
Snord22	100127111	small nucleolar RNA, C/D box 22	-1.48	9.40	36.60	1.45E-09	2.59E-06
Tmem38b	52076	transmembrane protein 38B	-2.03	12.86	34.79	3.68E-09	4.91E-06
Vaultrc5	378472	vault RNA component 5	-0.92	5.95	26.78	2.28E-07	0.00024
Snord15a	449630	small nucleolar RNA, C/D box 15A	-1.15	6.16	25.85	3.70E-07	0.00033
Snord17	100313519	small nucleolar RNA, C/D box 17	-1.35	7.30	24.17	8.82E-07	0.00067
Snora44	100217418	small nucleolar RNA, H/ACA box 44	-1.57	4.14	22.63	1.96E-06	0.00131
Sec14l4	103655	SEC14-like 4 (S. cerevisiae)	-1.47	3.85	20.22	6.91E-06	0.00410
Ctsf	56464	cathepsin F	4.13	7.18	19.27	1.13E-05	0.00606
Gpt	76282	glutamic pyruvic transaminase, soluble	-0.76	5.23	17.10	3.54E-05	0.01719
Rnu11	353373	U11 small nuclear RNA	-1.64	4.37	16.89	3.97E-05	0.01768
Eif5a	276770	eukaryotic translation initiation factor 5A	-3.72	9.08	14.91	0.000113	0.04645
5730408K05Rik	67531	RIKEN cDNA 5730408K05 gene	-1.07	4.80	14.70	0.000126	0.04799
**CD vs DRVO F0**							
Snord15b	449631	small nucleolar RNA, C/D box 14B	-1.29	7.57	41.63	1.10E-10	5.88E-07
Tbr1	21375	T-box brain gene 1	-2.20	4.46	36.99	1.19E-09	3.17E-06
Mir2137	100316779	microRNA 2137	2.69	4.52	36.13	1.84E-09	3.28E-06
Snord15a	449630	small nucleolar RNA, C/D box 15A	-1.31	6.16	35.22	2.95E-09	3.94E-06
A630072M18Rik	320770	RIKEN cDNA A630072M18 gene	-1.48	6.11	26.96	2.08E-07	0.00022
Gm20594	100463512	predicted gene, 20594	1.73	8.23	26.35	2.85E-07	0.00025
Phlpp1	98432	PH domain and leucine rich repeat protein phosphatase 1	-0.96	6.33	24.77	6.45E-07	0.00045
Fgr	14191	Gardner-Rasheed feline sarcoma viral (Fgr) oncogene homolog	-1.23	4.87	24.59	7.10E-07	0.00045
Grk4	14772	G protein-coupled receptor kinase 4	1.20	5.93	24.47	7.57E-07	0.00045
Rn45s	100861531	45S pre-ribosomal RNA	1.57	18.51	23.94	9.94E-07	0.00053
Snora78	100306952	small nucleolar RNA, H/ACA box 7	-1.03	6.53	22.14	2.54E-06	0.00123
Ube3c	100763	ubiquitin protein ligase E3C	-1.60	9.57	20.79	5.11E-06	0.00193
Cdk9	107951	cyclin-dependent kinase 9 (CDC2-related kinase)	-3.04	9.39	20.69	5.39E-06	0.00193
Mtap7d2	N/A	N/A	-3.21	4.84	20.69	5.40E-06	0.00193
Cadm4	260299	cell adhesion molecule 4	-2.71	5.68	20.69	5.41E-06	0.00193
Scarna2	100217438	small Cajal body-specific RNA 2	-0.82	5.87	20.09	7.37E-06	0.00246
A630089N07Rik	320586	RIKEN cDNA A630089N07 gene	1.16	6.77	19.44	1.04E-05	0.00326
Fam43b	625638	family with sequence similarity 43, member B	-1.32	5.06	19.16	1.20E-05	0.00357
Dact3	629378	dapper homolog 3, antagonist of beta-catenin (xenopus)	-1.08	8.14	19.00	1.31E-05	0.00367
Suv39h1	20937	suppressor of variegation 3-9 homolog 1 (Drosophila)	-0.92	5.87	18.90	1.38E-05	0.00369
Eif5a	276770	eukaryotic translation initiation factor 5A	-3.95	9.08	18.56	1.64E-05	0.00401
Mesdc1	80889	mesoderm development candidate 1	-1.45	5.34	18.54	1.66E-05	0.00401
Rab21	216344	RAB21, member RAS oncogene family	-2.53	6.30	18.47	1.73E-05	0.00401
Ssbp3	72475	single-stranded DNA binding protein 3	-2.30	5.41	18.37	1.82E-05	0.00405
4922505G16Rik	N/A	N/A	-1.45	3.95	18.23	1.96E-05	0.00419
Arhgap5	11855	Rho GTPase activating protein 5	0.87	6.56	17.95	2.27E-05	0.00467
Gm17821	100316870	DDB1 and CUL4 associated factor 5 pseudogene	1.03	7.97	17.62	2.70E-05	0.00528
Tead3	21678	TEA domain family member 3	-1.23	7.23	17.57	2.77E-05	0.00528
Kcnq1ot1	63830	KCNQ1 overlapping transcript 1	0.84	8.65	17.42	2.99E-05	0.00551
Tiam2	24001	T cell lymphoma invasion and metastasis 2	-1.28	3.93	17.35	3.11E-05	0.00554
Trim71	636931	tripartite motif-containing 71	-2.50	4.25	17.13	3.48E-05	0.00599
AI316807	N/A	N/A	-1.86	4.51	17.08	3.59E-05	0.00599

Banp	53325	BTG3 associated nuclear protein	-1.32	3.92	16.92	3.89E-05	0.00630
Foxg1	15228	forkhead box G1	-2.58	7.00	16.60	4.61E-05	0.00725
Slc16a6	104681	solute carrier family 16 (monocarboxylic acid transporters), member 6	-1.27	10.54	16.07	6.10E-05	0.00931
Beta-s	N/A	N/A	-1.57	6.26	15.90	6.68E-05	0.00981
Snord17	100313519	small nucleolar RNA, C/D box 17	-1.04	7.30	15.87	6.79E-05	0.00981
Mir5109	N/A	N/A	1.18	7.06	15.72	7.36E-05	0.01011
Zdhhc8	27801	zinc finger, DHHC domain containing 8	-1.12	4.95	15.71	7.38E-05	0.01011
Ptprm	19274	protein tyrosine phosphatase, receptor type, M	-0.75	5.90	15.62	7.75E-05	0.01035
Tlx3	27140	T cell leukemia, homeobox 3	-1.52	9.26	15.50	8.26E-05	0.01076
3110062M04Rik	78412	RIKEN cDNA 3110062M04 gene	-1.02	6.17	15.10	0.000102	0.01299
Dmp1	13406	dentin matrix protein 1	-1.22	10.62	15.04	0.000105	0.01305
Scaf1	233208	SR-related CTD-associated factor 1	-2.35	9.62	14.70	0.000126	0.01527
Ntn3	18209	netrin 3	-1.33	6.75	14.22	0.000163	0.01915
Zc3h15	69082	zinc finger CCCH-type containing 15	0.71	5.13	14.19	0.000165	0.01915
Piga	18700	phosphatidylinositol glycan anchor biosynthesis, class A	1.10	3.82	14.02	0.000181	0.02055
Cxxc4	319478	CXXC finger 4	-1.04	6.74	13.93	0.00019	0.02115
Nbea	26422	neurobeachin	-1.01	7.88	13.87	0.000195	0.02130
Fbxo10	269529	F-box protein 10	-0.97	4.37	13.83	0.0002	0.02138
Mir5126	100628595	microRNA 5126	-0.90	8.35	13.41	0.00025	0.02604
Csnk1a1	93687	casein kinase 1, alpha 1	0.61	5.84	13.39	0.000253	0.02604
Agap2	216439	ArfGAP with GTPase domain, ankyrin repeat and PH domain 2	-2.05	5.14	13.32	0.000262	0.02645
Tppp	72948	tubulin polymerization promoting protein	-1.04	3.96	12.99	0.000313	0.03094
Scyl2	213326	SCY1-like 2 (S. cerevisiae)	-1.02	5.02	12.77	0.000352	0.03418
Atxn10	54138	ataxin 10	0.70	5.19	12.58	0.00039	0.03720
Tbrg4	21379	transforming growth factor beta regulated gene 4	-0.89	4.22	12.41	0.000427	0.04006
Galnt10	171212	UDP-N-acetyl-alpha-D-galactosamine:polypeptide N-acetylgalactosaminyltransferase 10	-0.87	4.45	12.31	0.000451	0.04157
Memo1	76890	mediator of cell motility 1	-1.05	4.47	11.98	0.000537	0.04865
Smo	319757	smoothened homolog (Drosophila)	-0.87	4.42	11.93	0.000552	0.04918
**DR vs DRVO F0**							
Tmem38b	52076	transmembrane protein 38B	3.04	12.86	78.04	1.01E-18	5.40E-15
Rn45s	100861531	45S pre-ribosomal RNA	2.16	18.51	39.87	2.72E-10	6.94E-07
Casp9	12371	caspase 9	-1.51	5.97	39.16	3.90E-10	6.94E-07
Gm20594	100463512	predicted gene, 20594	2.15	8.23	37.92	7.35E-10	9.82E-07
Mir2137	100316779	microRNA 2137	2.62	4.52	33.89	5.84E-09	6.24E-06
Mir5109	N/A	N/A	1.69	7.06	31.23	2.29E-08	2.04E-05
Sec14l4	103655	SEC14-like 4 (S. cerevisiae)	1.56	3.85	27.29	1.75E-07	0.00013
Snord22	100127111	small nucleolar RNA, C/D box 22	1.08	9.40	21.33	3.87E-06	0.00237
Sox1	20664	SRY (sex determining region Y)-box 1	-1.08	9.70	21.27	4.00E-06	0.00237
3110062M04Rik	78412	RIKEN cDNA 3110062M04 gene	-1.19	6.17	20.89	4.87E-06	0.00260
Snord104	100216537	small nucleolar RNA, C/D box 104	1.96	5.20	19.33	1.10E-05	0.00505
Ctsf	56464	cathepsin F	-4.10	7.18	19.27	1.14E-05	0.00505
Dmp1	13406	dentin matrix protein 1	-1.37	10.62	19.05	1.27E-05	0.00523
Camk2b	12323	calcium/calmodulin-dependent protein kinase II, beta	1.03	4.70	16.92	3.91E-05	0.01492
Prkaa1	105787	protein kinase, AMP-activated, alpha 1 catalytic subunit	0.97	4.06	15.96	6.48E-05	0.02310
Snord49b	100217426	small nucleolar RNA, C/D box 49B	2.06	4.27	15.70	7.42E-05	0.02479
Egln1	112405	egl-9 family hypoxia-inducible factor 1	-0.78	5.12	14.62	0.000132	0.04144
Lars2	102436	leucyl-tRNA synthetase, mitochondrial	2.16	14.31	14.30	0.000156	0.04623

Legend for abbreviations/shading used in this table

Entrez ID: Gene ID as per the NCBI gene database

logFC: Log base 2 fold change between the two categories. Positive values indicate upregulation; negative values indicate downregulation

logCPM: Average Log base 2 transformed normalised gene counts over all samples

LR: Log rank.

p Value: Unadjusted (raw) p-vale from the log-rank test

FDR: False discovery rate (multiple testing correction)

Genes are similarly down-regulated in offspring pancreas by pateranl diet restriction; regardless of supplementation with vitamins and antioxidants

Genes are similarly either up- (3) or down- (2) regulated in offspring pancreas by pateranl supplementation with vitamins and antioxidants; regardless of diet restriction

Genes are similarly either up- (3) or down- (2) regulated in offspring pancreas by pateranl diet restriction;
-diet restriction and supplementation with vitamins and antioxidants has an opposing effect on the expression of the same gene (ie restored to control levels - see main Figure 4)

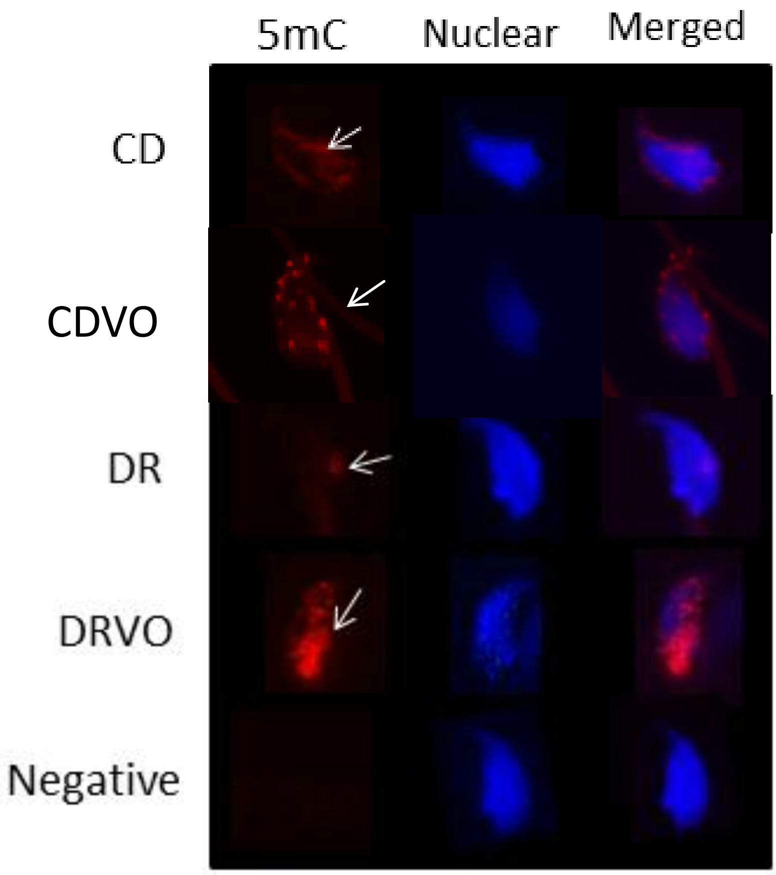

Figure S1: 5mC in founder sperm

Representative images of sperm positive for 5mC staining in the CD, CDVO, DR and DRVO founder groups. Arrows represent positive staining for 5mC. Red = 5mC, Blue = nuclear.

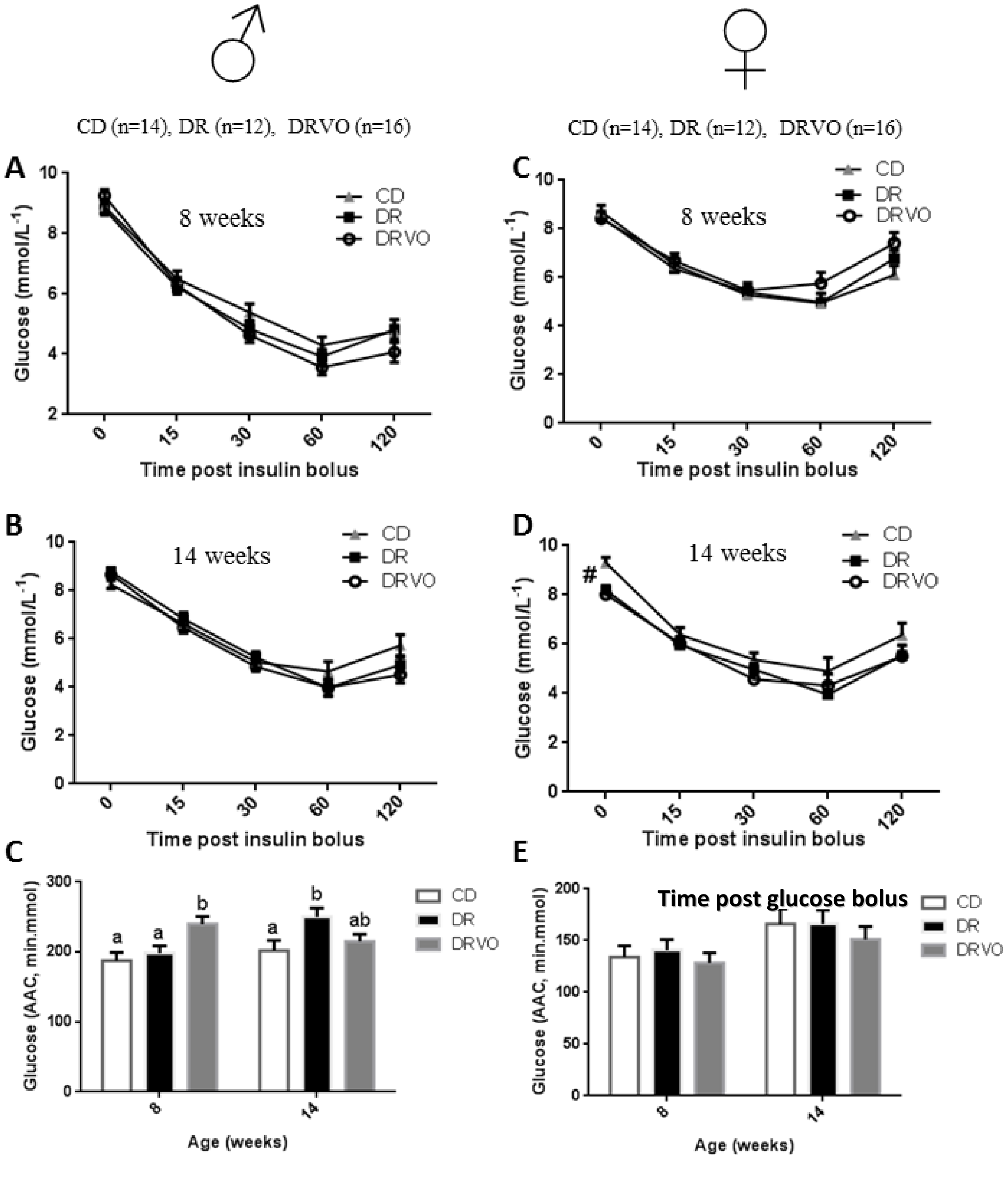

Figure S2: The effect of diet restriction and vitamin/antioxidant supplementation on offspring insulin tolerance

A Insulin tolerance as assessed by insulin tolerance test (ITT, 1.0 IU) for male offspring at 8 weeks **B** Insulin tolerance as assessed by insulin tolerance test (ITT, 1.0 IU) for male offspring at 14 weeks **C** Glucose area above the curve (AAC, min.mol) during ITT over age for male offspring **D** Insulin tolerance as assessed by insulin tolerance test (ITT, 0.75 IU) for

female offspring at 8 weeks **E** Insulin tolerance as assessed by insulin tolerance test (ITT, 0.75 IU) for female offspring at 14 weeks and **F** Glucose area above the curve (AAC, min.mmol) during ITT over age for female offspring.

Data represents 14 females and 14 males from 7 litters generated from 6 founders for CD group, 12 females and 12 males from 6 litters generated from 6 founders for DR group, and 16 females and 16 males from 8 litters generated from 8 founders for the DRVO group. Statistical significance was determined by repeated measures ANOVA (AUC) or general linear mixed model with founder ID added as a random factor.

Data is expressed as mean ± SEM.

Different letters denote significance at $P<0.05$.

# Denotes significant difference in fed baseline glucose levels ($mmol/L^{-1}$) between female offspring produced by CD founders compared with female offspring produced by DR and DRVO founders at $P<0.05$.

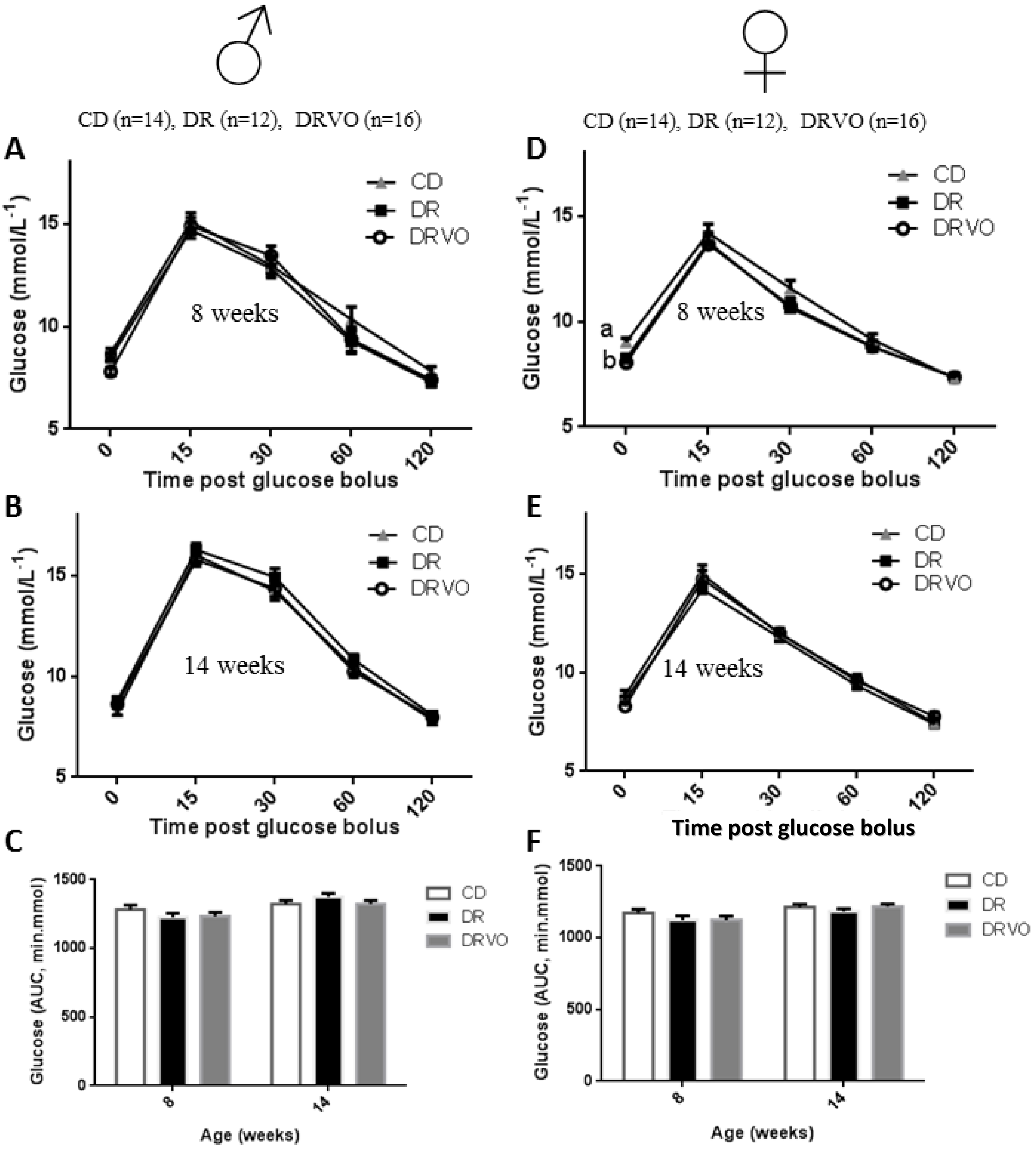

Figure S3: The effect of diet restriction and vitamin/antioxidant supplementation on offspring glucose tolerance

A Glucose tolerance as assessed by glucose tolerance test (GTT, 2g/kg) for male offspring at 8 weeks. **B** Glucose tolerance as assessed by glucose tolerance test (GTT, 2g/kg) for male offspring at 14 weeks. **C** Glucose area under the curve (AUC, min.mmol) during GTT over age for male offspring. **D** Glucose tolerance as assessed by glucose tolerance test (GTT,

2g/kg) for female offspring at 8 weeks. **E** Glucose tolerance as assessed by glucose tolerance test (GTT, 2g/kg) for female offspring at 14 weeks. **F** Glucose area under the curve (AUC, min.mmol) during GTT over age for female offspring.

Data represents 14 females and 14 males from 7 litters generated from 6 founders for CD group, 12 females and 12 males from 6 litters generated from 6 founders for DR group, and 16 females and 16 males from 8 litters generated from 8 founders for the DRVO group. Statistical significance was determined by repeated measures ANOVA (AAC) or general linear mixed model with founder ID added as a random factor.

Data is expressed as mean ± SEM.

Different letters denote significance at $P<0.05$.

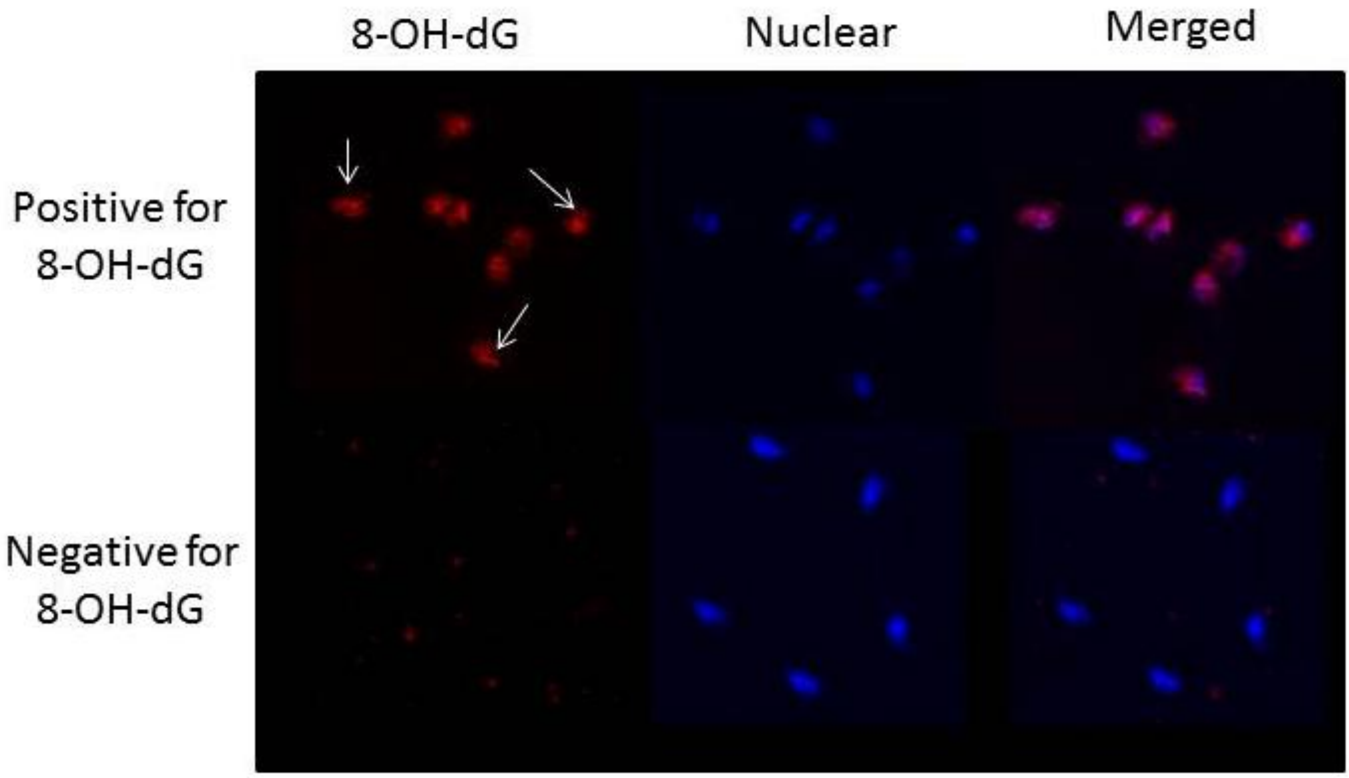

Figure S4: Scoring of 8-OHdG staining in founder sperm

Founder male sperm classified as stained positive for 8-OH-dG or sperm classified as stained negative for 8-OH-dG. Arrows represent positive sperm for 8-OH-dG co-localised with sperm nucleus. Red = 8-OH-dG, blue = Nuclear.
